# New insights into the blood-stage transcriptome of *Plasmodium falciparum* using RNA-Seq

**DOI:** 10.1111/j.1365-2958.2009.07026.x

**Published:** 2010-02-05

**Authors:** Thomas D Otto, Daniel Wilinski, Sammy Assefa, Thomas M Keane, Louis R Sarry, Ulrike Böhme, Jacob Lemieux, Bart Barrell, Arnab Pain, Matthew Berriman, Chris Newbold, Manuel Llinás

**Affiliations:** 1 Parasite Genomics, Wellcome Trust Sanger Institute, Wellcome Trust Genome CampusHinxton, Cambridge, CB10 1SA, UK; 2 Department of Molecular Biology and Lewis-Sigler Institute for Integrative Genomics, Princeton UniversityPrinceton, NJ 08544, USA; 3 University of Oxford, Weatherall Institute of Molecular Medicine, John Radcliffe HospitalHeadington, Oxford OX3 9DS, UK

## Abstract

Recent advances in high-throughput sequencing present a new opportunity to deeply probe an organism's transcriptome. In this study, we used Illumina-based massively parallel sequencing to gain new insight into the transcriptome (RNA-Seq) of the human malaria parasite, *Plasmodium falciparum*. Using data collected at seven time points during the intraerythrocytic developmental cycle, we (i) detect novel gene transcripts; (ii) correct hundreds of gene models; (iii) propose alternative splicing events; and (iv) predict 5′ and 3′ untranslated regions. Approximately 70% of the unique sequencing reads map to previously annotated protein-coding genes. The RNA-Seq results greatly improve existing annotation of the *P. falciparum* genome with over 10% of gene models modified. Our data confirm 75% of predicted splice sites and identify 202 new splice sites, including 84 previously uncharacterized alternative splicing events. We also discovered 107 novel transcripts and expression of 38 pseudogenes, with many demonstrating differential expression across the developmental time series. Our RNA-Seq results correlate well with DNA microarray analysis performed in parallel on the same samples, and provide improved resolution over the microarray-based method. These data reveal new features of the *P. falciparum* transcriptional landscape and significantly advance our understanding of the parasite's red blood cell-stage transcriptome.

## Introduction

*Plasmodium falciparum* malaria is responsible for more than one million deaths annually, most of which occur in young children (Breman, 2001). For several decades, the development of new antimalarial compounds has been slow, mostly due to a lack of well-defined *Plasmodium*-specific targets, adding to a growing concern as established drugs become ineffective due to widespread resistance in the field (Arav-Boger and Shapiro, 2005). In 2002, the genome of the 3D7 clone of *P. falciparum* was sequenced (Gardner *et al.*, 2002), renewing hope that progress towards reducing the burden of malaria would be greatly accelerated. The *P. falciparum* genome encodes roughly 5400 genes and has the lowest G+C content (19%) of any genome sequenced to date. Approximately half of the predicted coding sequences (CDSs) are uncharacterized, with little sequence similarity outside the *Plasmodium* genus, and a large number of genes and gene families are unique to *P. falciparum*. Furthermore, the proteome contains a high proportion of low complexity sequence where poly-asparagine regions are highly prevalent (Aravind *et al.*, 2003).

Making use of these sequence data, two groups in 2003 used different DNA microarray platforms (70-mer oligonucleotide based, and Affymetrix) to measure transcript levels during the intraerythrocytic developmental cycle (IDC) of the *P. falciparum* parasite. These studies revealed a highly ordered cascade of gene expression (Bozdech *et al.*, 2003; Le Roch *et al.*, 2003). A later study demonstrated that gene expression levels were similar between different strains of disparate geographical origin, suggesting highly conserved modes of transcriptional regulation during development (Llinas *et al.*, 2006). More recently gene expression studies in a variety of other life cycle stages have been performed (Young *et al.*, 2005; Siau *et al.*, 2008; Tarun *et al.*, 2008). Previous efforts to sequence RNA transcripts from *P. falciparum* have focused on expressed sequence tags (Lu *et al.*, 2007) or have analysed a small fraction of the full-length cDNA sequences from the *Plasmodium* spp. (Wakaguri *et al.*, 2009a,b;). These low-resolution studies already demonstrated inaccuracies in *P. falciparum* gene model predictions and suggested that both variable length untranslated regions (UTRs) and diversity in splicing were prevalent in the transcriptome.

The depth of sequence obtainable with highly parallel sequencing technologies such as Illumina's Genome Analyzer (Bentley *et al.*, 2008), 454 (Droege and Hill, 2008), SOLiD (Ondov *et al.*, 2008) (http://solid.appliedbiosystems.com) platforms make it possible to sequence cDNA and obtain high coverage of all transcribed genes. In 2008, the first applications of high-throughput sequencing technologies to the direct sequencing of expressed RNA transcripts (RNA-Seq) from human tissue (Pan *et al.*, 2008; Sultan *et al.*, 2008), yeast (Nagalakshmi *et al.*, 2008; Wilhelm *et al.*, 2008) and mouse (Mortazavi *et al.*, 2008) were reported. RNA-Seq can reliably be used to correct gene annotations, confirm new and existing splice forms, analyse UTR regions, define non-coding RNAs or find new transcripts (Wang *et al.*, 2009). In general, samples for RNA-Seq are produced by reverse transcription of purified mRNA using oligo(dT) and random priming. The sequencing results, typically short reads between 25 and 75 bp, as either singletons or as paired-end reads can then be mapped onto a reference genome with computational tools such as SSAHA2 (Ning *et al.*, 2001), MAQ (Li *et al.*, 2008), BWA (Li and Durbin, 2009) and ELAND (http://www.illumina.com/). From the mapping position of each read, the cumulative occurrence per base pair can be calculated to generate genome coverage plots.

In this study, we applied RNA-Seq to seven time points from the asexual IDC of *P. falciparum* with the aim of capturing features associated with all expressed RNA transcripts and measuring splicing dynamics that occur during parasite development. Despite the high A+T content of the genome, which presents challenges for mapping transcripts, we were able to detect transcription of 4871 genes during the 48 h IDC. While a previous report had demonstrated the feasibility of the short read sequencing approach for *P. falciparum*, these results were based on sequencing a biased mRNA sample prepared using an oligo(dT) affinity strategy (Sorber *et al.*, 2008). Our methodology provides an improvement in genome-wide sequence coverage due to an enhanced enrichment strategy for mRNAs. Overall, our results indicate a higher level of transcription than previously realized by DNA microarray studies due to our ability to detect far more low abundance species. We have identified over 100 new transcripts in the genome and reannotated more than 10% of the existing gene models. Finally, we were able to identify numerous alternative splicing events and further our understanding of 5′ and 3′ UTRs, which may lend further insight into gene regulation in this important human pathogen.

## Results

### Comparison of depletion methods

Due to the high abundance of ribosomes in all cells, ribosomal RNAs (rRNAs) account for over 90% of all cellular RNAs. Therefore, the most standard method for rRNA depletion is to affinity purify polyadenylated mRNA species using an oligo(dT) sepharose. For *P. falciparum*, this methodology does not work effectively because of the high A+T content of the genome (80% in coding and 90% in non-coding regions). In previous DNA microarray-based genome-wide transcriptome analyses, this effect has largely been ignored with cDNA being synthesized directly from total RNA using oligo(dT) followed by *in vitro* reverse transcription (Chen *et al.*, 2003) or a combination of oligo(dT) and random priming (Bozdech *et al.*, 2003). Either of these methods results in an over-representation of parasite rRNAs in the material assayed on the microarray but, in general, does not compromise the performance of the microarray. However, for a sequence-based approach, removal of abundant rRNA is essential in order to maximize coverage of other low expressed transcripts, because the total number of possible reads per machine run is finite.

In an attempt to remove a significant fraction of plasmodial rRNAs, we tested two possible mRNA enrichment strategies. The first method is affinity-based and targets 26 high abundance rRNA species as well as the 32 most abundant mRNA species in *P. falciparum* such as the histones, merozoite surface protein 1 and several heat shock proteins (based on previous microarray data sets) (Bozdech *et al.*, 2003; Chen *et al.*, 2003) (see Table S1). To accomplish this, we first bound 58 biotin-tagged DNA oligonucleotides to magnetic streptavidin beads and then incubated isolated total RNA in the presence of the immobilized (complementary) DNA sequences. Subsequently, the unbound RNAs were isolated and subjected to reverse transcription for Illumina sequencing (see *Experimental procedures*). Our second method was to treat with Terminator™ 5′-Phosphate-Dependent Exonuclease (Epicentre) (a processive 5′-3′ exonuclease that digests RNA containing a free 5′-monophosphate end, thereby removing rRNAs, transfer RNAs, and apicoplast-derived RNAs) and then to affinity deplete with our 58 biotin-tagged DNA oligonucleotides. Independent sequencing runs were performed to compare the effectiveness of these approaches (Table 1, Table S1). Although comparable numbers of reads (> 4.5 million) were attained for all three samples, 60% of the reads from the undepleted sample mapped against rRNA loci targeted by our depletion strategies and covered only 1% of the genome more than 10 times. However, using the affinity oligonucleotide depletion or combined oligonucleotide and Terminator™ depletion strategies, 58% and 21%, respectively, mapped to the depleted regions. Furthermore, the percentage of the genome with more than 10× coverage increased to 2% and 7% respectively, and overall genome coverage increased more than five times over the undepleted sample. Finally, the genome coverage is overall much greater in the depleted samples and is maximal (51%) in the combined Terminator™ and oligonucleotide-depleted sample. Based on these results, we conclude that for enrichment of *P. falciparum* mRNAs for high-throughput sequencing, the combined oligonucleotide and Terminator™ exonuclease depletion strategy was the best. We therefore used a combined method of Terminator™ exonuclease depletion in conjunction with the biotin-oligonucleotide depletion strategy for all subsequent experiments.

**Table 1:** RNA-Seq mapping statistics against *P. falciparum* genome.

	Sequencing run		
	Undepleted	Depleted by specific oligos	Depleted by exonuclease and specific oligos
Total reads	5 161 203	5 657 762	4 847 379
% Mapped^a^	94	92	86
% Mapped to unique locations^a^	15	24	52
Reads mapped to rRNA	3 120 248	3 269 563	1 034 004
% Reads mapped to rRNA	60%	58%	21%
Fold coverage	1.14	2.05	3.75
% Genome not covered	72	65	49
% Genome covered, < 5-fold	96	93	84
% Genome covered, > 10-fold	1	2	7
Max. coverage across in coding sequences^b^	11 008	4 697	7 061
Max. coverage, genome-wide^b^	73 864	95 351	64 552
Max. average coverage in coding sequences^b^	2 774	1 296	1 598
Genes with gmean coverage > 5^b^	749	1 205	2 438
Genes with gmean coverage > 10^b^	93	191	499

Summary statistics of mapping of Illumina sequencing reads on to *P. falciparum* 3D7 genome from RNA-Seq runs after depletion by specific oligonucleotides and by exonuclease digestion. Oligonucleotides used for specific depletion have been described in Table S1.

a Reads mapped using SSAHA2.

b Coverage determined using MAQ; non-unique reads randomly partitioned over repeats.

### Sequencing and data processing

**Fig. 1:**
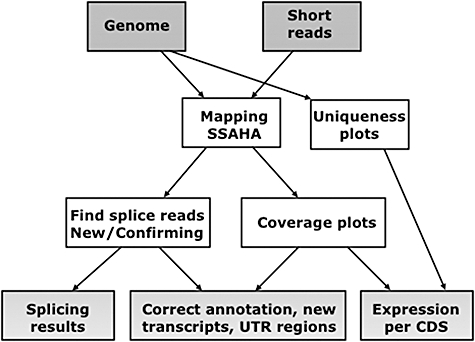
Workflow of short read processing for gene expression analysis by RNA-Seq. The Illumina sequencing reads are mapped with SSAHA2 (Ning *et al.*, 2001) against the *Plasmodium falciparum* 3D7 genome. After mapping, splice reads and coverage plots are obtained. The splice reads are used to confirm or find new splice sites as well as alternative splice sites. The coverage plots show the RNA expression levels over each base pair of the genome. To calculate the expression per CDS per time point, the coverage plots and the uniqueness plots are used. Uniqueness plots indicate the uniqueness of a particular region of the genome. Using the coverage, it is possible to identify incorrect annotation, novel transcripts and potential untranslated regions (UTRs) of protein coding transcripts (as described in the text).

Using highly synchronized 3D7 parasites, we collected RNA samples at seven different time points every 8 h for 48 h, thus capturing the entire IDC of *P. falciparum* from the ring stage to mature schizonts. Total RNA samples were processed as described above and cDNA generated by reverse transcription using a 1:1 combination of oligo(dT) and random nonamer primers (see *Experimental procedures*). Illumina sequencing reads were mapped and processed using standard methods developed at the Sanger Institute (see Fig. 1, *Experimental procedures*). Complete mapping statistics are presented in Table S2. In summary, between 51% (early rings) and 20% (late schizonts) of the reads mapped uniquely against the genome. Of these, around 50–60% of the reads mapped to predicted protein-coding genes and 20% mapped within 1.0 kb up- or downstream of genes, demarcating possible UTRs. For each individual time point, we calculated a transcript abundance value based on the geometric mean depth of coverage (see below) of all sequencing reads corresponding to a given genomic region (see *Experimental procedures*). These mapped regions include previously annotated genes (Table S3), annotated pseudo genes (Table S4) and novel genes predicted by our results (Table S5). The temporal data capture the dynamic variation of mRNA abundance values during the IDC and correlate well with previous microarray studies (see below). The total range of transcriptional activity captured by these data varied by five orders of magnitude. Using data from CDS only, variation in gene expression by a factor of up to 8200 was observed.

### Correlations to DNA microarray data

**Fig. 2:**
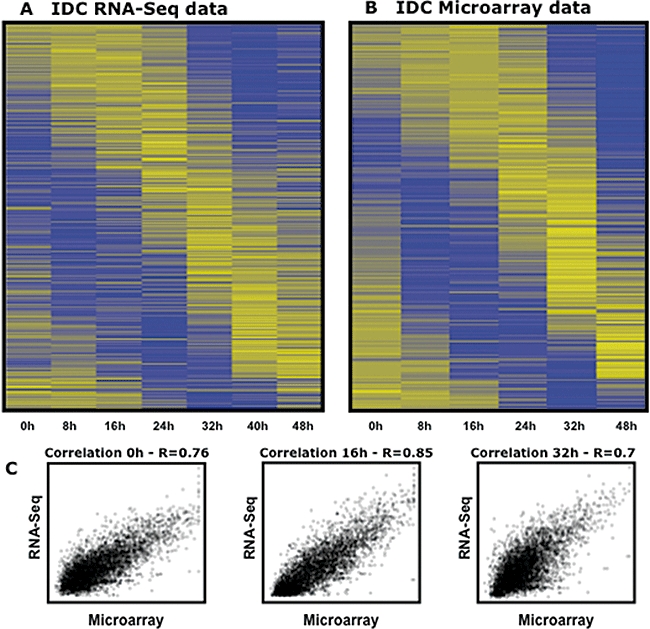
Expression profiles of 3975 annotated genes at seven time points in the intra-erythrocytic developmental cycle (IDC) of *P. falciparum* 3D7 and comparison of RNA-Seq data with microarray data. A. Heat map of genes expressed in the IDC cycle (Bozdech *et al.*, 2003) with the RNA-Seq data. B. Heat map of genes expressed in the IDC cycle, derived from microarray experiments using the identical biological samples. C. Pearson correlation between the RNA-Seq and the microarray data sets.

As these are the first temporal, sequencing-based transcriptome data generated from *P. falciparum* mRNAs during the IDC, we wanted to directly compare and validate our sequencing results with microarray data generated in parallel using the same RNA samples. Previous studies have found variable correlations when measuring gene expression with different technologies (Bloom *et al.*, 2009), suggesting that both methods have their biases when compared with quantitative PCR. When plotted according to the phase of gene expression, as previously determined by Llinas *et al.* (2006), the well-established cascade of gene expression is faithfully reproduced by both data sets (Fig. 2A and B). The RNA-Seq and DNA microarray data are in good agreement, with Pearson correlations between these data sets ranging from 0.7 to 0.85 at various time points (Fig. 2C, Table S2).

In addition to the high correlation with microarray data, we estimated the hours post invasion (hpi) for each time point of the RNA-Seq in the IDC using a statistical likelihood-based estimator that calculates the relative temporal sequence and a generalized overview expression curve for each time point (Fig. S1) (Lemieux *et al.*, 2009). These data recapitulate the systematic temporal progression of parasite development during this experiment and demonstrate that the cultures are well synchronized.

### Transcriptome analysis

**Fig. 3:**
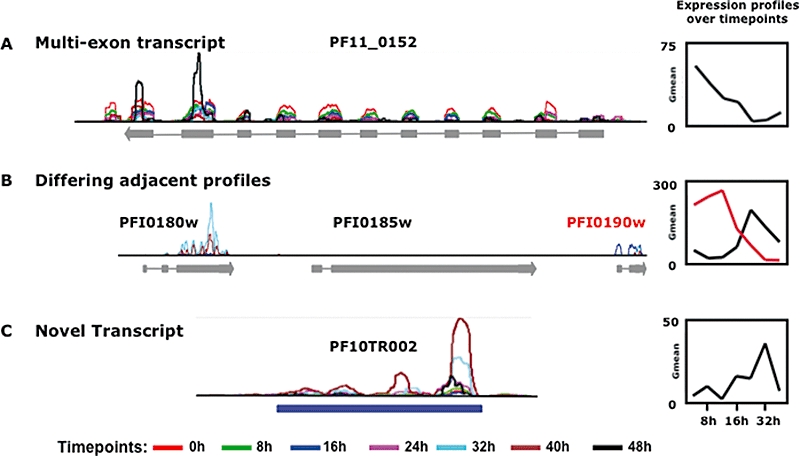
RNA-Seq coverage plots for selected genes and their corresponding expression profiles (expressed as gmean) at seven time points in the intra-erythrocytic developmental cycle (IDC) of *P. falciparum* 3D7. A. Expression profile of a multi-exon gene PF11_0152 (GTPase activator) [maximal expression 423 (gmean)]. B. Expression profiles of three adjacent genes: PFI0180w (max expression 2000), PFI0185w (no expression) and PFI0190w (max expression 780) on chromosome 9, showing opposite temporal regulation of expression for PFI0180w (alpha tubulin – black profile plot), PFI0190w (60S ribosomal protein L32 – red profile plot) and lack of expression for PFI0185w. C. Expression profile of a novel mlncRNA transcript (PF10TR002, see in Table S5) identified on Chr10 (max expression 580).

Our RNA-Seq data capture approximately 90% of the predicted CDS in the *P. falciparum* genome. As the data are highly variable across the length of a transcript, for each predicted CDS locus in GeneDB (http://www.genedb.org/), we have calculated an mRNA abundance value based on the geometric mean of the sequencing reads (Table S3). Many *P. falciparum* genes contain low complexity sequence which means that reads cannot always be unambiguously aligned to these regions. In order to correct for this mapping error we have adjusted the geometric mean, using as a denominator the region of each gene that we calculate to be mappable (see *Experimental procedures*). It is notable that the percentage of uniquely mapping reads is lower at the schizont stage than at other time points. This may be explained both by the fact that this set of reads were 37 bp rather than 54 bp (and so were less likely to map uniquely) and to the observation that the proportion of proteins containing low complexity sequence appears higher towards the end of the IDC (data not shown). The data can easily be used to discriminate exons within a given gene and to identify novel transcripts (Fig. 3). Even for highly complex intron/exon structures such as that seen for the GTPase activator (PF11_0152), a coding gene that is comprised of 11 exons, we can easily confirm the predicted splice sites (Fig. 3). Moreover, adjacent genes with opposite temporal expression profiles can be observed using the RNA-Seq data. For example, PFI0180w (alpha tubulin) is maximally expressed during the trophozoite stage, while the next gene PFI0185w is not expressed, while the following downstream gene, PFI0190w is a ring stage RNA (Fig. 3).

**Fig. 4:**
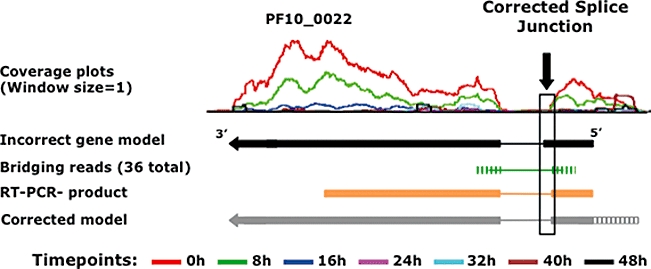
Use of RNA-Seq data in correction of gene models in *P. falciparum* 3D7. An example is shown where a previously incorrect predicted gene model was corrected using RNA-Seq evidence for the gene PF10_0022 [*Plasmodium* exported protein (PHISTc)]. The coverage plots indicate that the first exon is shorter by 27 bp at the 3′ end. The arrow and black boxed areas highlight the location of structural changes incorporated in the gene PF10_0022. The correctly spliced form is confirmed by 36 known bridging reads (green features). This new splice site was confirmed by RT-PCR (orange features). Coverage plots also identified the 5′ UTR in PF10_0022 (shown by grey striped feature). The incorrect gene model was taken from the published version of the *P. falciparum* 3D7 genome (Gardner *et al.*, 2002).

For some sequenced transcripts, putative UTRs can be discerned (Figs 3 and 4). However, in general the depth of sequencing coverage of the UTRs is lower compared with the exonic regions and is mostly attributable to the poor mapping of the relatively short Illumina reads onto the extremely high A+T-rich (> 90%) UTR regions. Unfortunately, this RNA-Seq data set does not serve to capture short non-protein coding transcripts previously predicted by several groups (Chakrabarti *et al.*, 2007; Mourier *et al.*, 2008; Mishra *et al.*, 2009) because we used a fragment size cut-off around 200 bp, excluding possible short RNAs.

### Correction of gene models

The RNA-Seq data are highly informative for correcting structural boundaries of predicted genes (Table 2). The most common correction was elongation or shortening of predicted intron/exon boundaries, in which case, the coverage plots of all expressed time points unambiguously drop towards zero before the splice site. For example, we predicted a shortened first exon for PF10_0022, a member of the PHISTc exported protein family (Sargeant *et al.*, 2006) (Fig. 4) and confirmed this experimentally by RT-PCR and directed sequencing of the PCR product across the splice junction (Fig. S2). Using the RNA-Seq data, we performed a genome-wide update of all *P. falciparum* splice sites, incorporating mostly data from this study but also changes due to homology to other recently sequenced species (Carlton *et al.*, 2008; Pain *et al.*, 2008) or expressed sequence tag evidence (Haas *et al.*, 2008). In all, 423 genes were modified (8% of the total predicted genes) 202 splice sites for new exons added, with the majority being confirmed by two or more RNA-Seq read pairs. Despite low coverage of UTRs, we were able to identify several gene models with strong UTR signals, and report 192 spliced UTR events (with at least two confirming reads).

**Table 2:** Overview of changes to annotation of the *P. falciparum* 3D7 genome.

	Previous annotation^a^	Modified annotation^b^	Difference
Predicted protein coding genes	5 317	5 438	121
Changes to gene structures based on RNA-Seq evidence		423	
Predicted spliced transcripts	2 870	2 952	82
Predicted splice sites	8 315	8 517	202
Confirmed splice sites (by ≥ 1 Illumina read pair)	6 590	6 891	301
Confirmed splice sites (by ≥ 2 Illumina read pairs)	6 095	6 389	294
% confirmed splice sites (by ≥ 2 Illumina reads pairs)	73	75	2
Reads confirming predicted splice sites	453 881	479 011	25 130

Overview of annotation changes in the *P. falciparum* 3D7 genome with the aid of the RNA-Seq data during the period between March, 2008 and May, 2009.

a Annotation from May 2008, produced without using RNA-Seq data.

b Annotation from March 2009, edited using RNA-Seq data.

Splicing is highly prevalent throughout the *P. falciparum* transcriptome. We used our RNA-Seq data to perform a genome-wide analysis of splicing and splice-site variants to identify a total of 8496 splice sites in 3D7. As expected, our data confirmed that the majority of splice sites in the genome were previously properly predicted. However, our data also serve to correct mispredicted splice sites and identify new and alternative splice sites. For splice site analysis, we used partially mapping reads (bridging reads), where two segments of the sequencing read map to different positions along the same chromosome but are anchored in the genome by the other end of the read pair. For new splice site reads, only a portion of the sequence maps to the CDS sequence, calling for re-analysis of the non-mapping segments (see *Experimental procedures*). In total, 294 possible new splice sites containing canonical splice donors and acceptors were confirmed by at least two RNA-Seq reads (Table 2). All new gene model predictions are available through GeneDB and have been deposited at http://PlasmoDB.org version 6.0 (Bahl *et al.*, 2003).

### Alternative splice sites

**Fig. 5:**
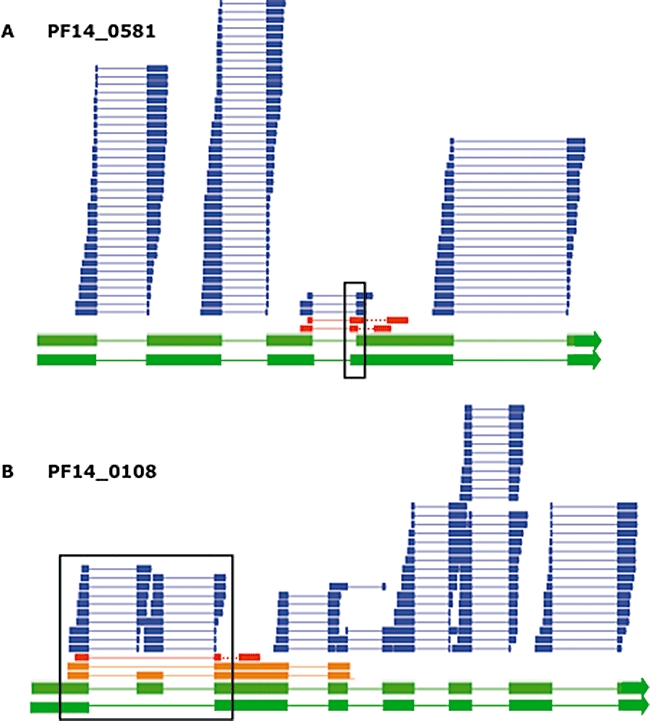
Use of RNA-Seq data to detect alternative splicing and exon skipping events in the IDC transcriptome of *P. falciparum* 3D7. A. Alternative splice sites for exon 4 of PF14_0581 (putative apicoplast ribosomal protein isoforms) highlighted by aligned bridging reads (red) from early ring time points. The boxed area highlights the location of alternative splicing in the gene PF14_0581. The dotted red line links read pairs from the same template DNA. The blue bars show reads that map to the borders of exons, across an intron. Perfectly mapping reads are not shown. B. Example of exon skipping in PF14_0108 (a predicted protein of unknown function). A new splice form was indicated by a read mapping across two introns and exon, and its read pair (red). Both splice-forms were confirmed by RT-PCR (orange boxes). The boxed area highlights the location of exon skipping in the gene PF14_0108. Only the last eight exons of PF14_0108 are shown in the figure.

Using reads spanning existing and new splice sites, we built an algorithm to identify alternative splicing (see *Experimental procedures*). We identified four types of alternative splice events: exon-skipping; intron retention/creation; 3′ and 5′ alternative splicing (coordinate changes to external exons) and 3′ and 5′ splicing that results in an alternative start or stop codon (Table 3). Out of a total of 84 alternative splice events, the most common is the 3′ and 5′ alternative splicing (56 events), followed by exon skipping (16 events). We required at least two confirming reads for a new splice site to be automatically assigned. All predictions were manually verified and included in the GeneDB database. In six instances (PFF0630c, PFI0280c, PF10_0149, MAL13P1.144, MAL13P1.267, MAL13P1.277) we found more than one alternative splicing event in a single gene. Figure 5A shows an example of two 5′ alternative splicing events for PF14_0581 (putative apicoplast ribosomal component). The new splice site occurs mostly in the 16 and 24 hpi samples, whereas the previously known splice site occurs in the later time points. This observation brings up the tantalizing possibility that alternative splicing is temporally regulated during the *P. falciparum* lifecycle. Overall, we did not detect a temporal bias for alternative splicing at any specific time point. In another case of alternative splicing in the PF14_0108 gene (unknown function), exon skipping was detected. Both transcripts were validated by RT-PCR and sequencing (Fig. S2).

**Table 3:** Overview of alternative splicing events confirmed by RNA-Seq.

Chr.	New splice site	Number of confirming reads	Alternative splicing events	Gene identifier	Product
1	294554.. 294928	3	Exon skipping	PFA0345w	Centrin-1
1	388453.. 388750	4	Exon skipping	PFA0485w	Phosphatidate cytidylyltransferase, putative
2	140595.. 140820	2	3′ and 5′ alternative	PFB0140w	Zinc finger protein, putative
2	231909.. 232099	10	3′ and 5′ alternative	PFB0255w	Conserved Plasmodium protein, unknown function
2	382875.. 383071	2	3′ and 5′ alternative	PFB0410c	Phospholipase A2, putative
2	412537.. 412701	3	3′ and 5′ alternative	PFB0455w	60S ribosomal protein L37ae, putative
2	276441.. 276558	5	Alternative stop	PFB0305c	Merozoite surface protein 5
3	214831.. 215062	3	3′ and 5′ alternative	PFC0200w	60S Ribosomal protein L44, putative
3	458474.. 458623	3	3′ and 5′ alternative	PFC0441c	SAC3/GNAP family-related protein, putative
3	553204.. 553287	13	3′ and 5′ alternative	PFC0571c	Conserved Plasmodium protein, unknown function
3	566965.. 567142	6	3′ and 5′ alternative	PFC0582c	Vesicle transport v-SNARE protein, putative
4	99991.. 100185	33	3′ and 5′ alternative	PFD0070c	rifin
4	673403.. 673580	2	3′ and 5′ alternative	PFD0720w	Conserved ARM repeats protein, unknown function
4	785847.. 785932	3	3′ and 5′ alternative	PFD0850c	Memo-like protein
5	1082779.. 1082882	2	3′ and 5′ alternative	PFE1305c	ADP-ribosylation factor GTPase-activating protein, putative
5	1147532.. 1147829	4	Exon skipping	PFE1375c	Conserved Plasmodium protein, unknown function
5	1202254.. 1202495	5	Intron creation	PFE1465w	Conserved Plasmodium protein, unknown function
6	1171453.. 1171616	2	3′ and 5′ alternative	PFF1375c	Ethanolaminephosphotrans ferase, putative
6	258869.. 258956	24	Intron creation	PFF0300w	RNA binding protein, putative
6	533969.. 534599	18	3′ and 5′ alternative	PFF0630c	Conserved Plasmodium protein, unknown function
6	533969.. 534618	42	Exon Skipping	PFF0630c	Conserved Plasmodium protein, unknown function
6	534448.. 534599	2	3′ and 5′ alternative	PFF0630c	Conserved Plasmodium protein, unknown function
6	534448.. 534618	6	3′ and 5′ alternative	PFF0630c	Conserved Plasmodium protein, unknown function
6	794834.. 794959	38	3′ and 5′ alternative	PFF0920c	Conserved Plasmodium protein, unknown function
7	80499.. 80835	3	Alternative stop	MAL7P1.2 25	Plasmodium exported protein (PHISTa-like), unknown function
7	112961.. 113197	2	3′ and 5′ alternative	MAL7P1.2 29	Cytoadherence linked asexual protein
7	137990.. 138206	2	3′ and 5′ alternative	PF07_0004	Plasmodium exported protein, unknown function
7	1314328.. 1314516	21	3′ and 5′ alternative	MAL7P1.1 60	Conserved Plasmodium protein, unknown function
8	553020.. 553217	11	3′ and 5′ alternative	MAL8P1.1 06	Conserved Plasmodium protein, unknown function
8	227245.. 227377	19	3′ and 5′ alternative	MAL8P1.1 43	Conserved Plasmodium protein, unknown function
8	284523.. 284611	2	3′ and 5′ alternative	MAL8P1.1 38	Alpha/beta hydrolase, putative
9	115387.. 115522	18	3′ and 5′ alternative	PFI0125c	Serine/Threonine protein kinase, FIKK family
9	285483.. 285587	59	3′ and 5′ alternative	PFI0280c	Autophagocytosis-associated protein, putative
9	285483.. 285808	3	Exon skipping	PFI0280c	Autophagocytosis-associated protein, putative
9	169401.. 169747	3	Alternative stop	PFI0175w	Conserved Plasmodium protein, unknown function
9	527195.. 527472	2	Exon skipping	PFI0560c	Conserved Plasmodium protein, unknown function
9	749906.. 750048	2	3′ and 5′ alternative	PFI0890c	Organelle ribosomal protein L3 precursor, putative
9	857945.. 858202	18	Exon skipping	PFI1030c	Ubiquitin conjugating enzyme, putative
9	1135552.. 1135898	8	3′ and 5′ alternative	PFI1375w	Cytochrome C oxidase, putative
9	1219040.. 1219282	2	3′ and 5′ alternative	PFI1490c	Ran-binding protein, putative
9	1427723.. 1427820	7	Alternative start	PFI1740c	Ring-exported protein 2
10	122332.. 122507	7	3′ and 5′ alternative	PF10_0028	RNA binding protein, putative
10	616830.. 617522	2	3′ and 5′ alternative	PF10_0149	Cysteinyl-tRNA synthetase, putative
10	617363.. 617522	5	3′ and 5′ alternative	PF10_0149	Cysteinyl-tRNA synthetase, putative
10	630332.. 630635	3	Exon skipping	PF10_0153a	Conserved Plasmodium protein, unknown function
10	1506427.. 1506546	1194	3′ and 5′ alternative	PF10_0372	Antigen UB05
11	204951.. 205102	9	3′ and 5′ alternative	PF11_0058	RNA polymerase subunit, putative
11	539968.. 540166	218	3′ and 5′ alternative	PF11_0149	Rhomboid protease ROM1, putative
11	616746.. 616900	4	3′ and 5′ alternative	PF11_0169	SNO glutamine amidotransferase, putative
11	736961.. 737116	3	3′ and 5′ alternative	PF11_0202	Clathrin coat assembly protein, putative
11	1029280.. 1029486	14	3′ and 5′ alternative	PF11_0273	DNAJ protein, putative
11	1431895.. 1433153	3	Alternative stop	PF11_0377	Casein kinase 1, PfCK1
12	194966.. 195139	2	3′ and 5′ alternative	PFL0190w	Ubiquitin conjugating enzyme E2, putative
12	545253.. 545453	2	3′ and 5′ alternative	PFL0610w	Conserved Plasmodium protein, unknown function
12	556129.. 556311	9	3′ and 5′ alternative	PFL0623c	conserved Plasmodium membrane protein, unknown function
12	675551.. 675934	15	Exon skipping	PFL0825c	Conserved Plasmodium protein, unknown function
12	848566.. 848672	4	3′ and 5′ alternative	PFL1015w	Conserved Plasmodium protein, unknown function
12	1429555.. 1429746	5	Intron creation	PFL1650w	Conserved Plasmodium protein, unknown function
13	598721.. 598890	3	3′ and 5′ alternative	MAL13P1. 70	Conserved Plasmodium membrane protein, unknown function
13	656337.. 656495	2	3′ and 5′ alternative	MAL13P1. 82	Phosphatidylinositol synthase
13	656725.. 657067	3	Exon skipping	MAL13P1. 82	Phosphatidylinositol synthase
13	670259.. 670473	22	3′ and 5′ alternative	MAL13P1. 84	Protein kinase, putative
13	892554.. 892819	3	3′ and 5′ alternative	MAL13P1. 118	3′,5′-cyclic nucleotide phosphodiesterase
13	1097484.. 1097716	2	Intron creation	MAL13P1. 144	Translation initiation factor EIF-2B gamma subunit, putative
13	1097497.. 1097716	2	Intron creation	MAL13P1. 144	Translation initiation factor EIF-2B gamma subunit, putative
13	1280278.. 1280427	5	3′ and 5′ alternative	MAL13P1. 163	ER lumen protein retaining receptor 1, putative
13	2037484.. 2037617	5	3′ and 5′ alternative	MAL13P1. 257	Conserved Plasmodium protein, unknown function
13	2093143.. 2093239	2	Intron creation	MAL13P1. 267	conserved Plasmodium protein, unknown function
13	2094689.. 2094839	5	Intron creation	MAL13P1. 267	Conserved Plasmodium protein, unknown function
13	2242140.. 2242247	3	Exon skipping	MAL13P1. 277	DNAJ-like protein, putative
13	2242140.. 2242464	4	Exon skipping	MAL13P1. 277	DNAJ-like protein, putative
13	2438774.. 2438947	88	3′ and 5′ alternative	MAL13P1. 303	Polyadenylate-binding protein, putative
13	2463514.. 2463766	10	Exon skipping	MAL13P1. 306	Conserved Plasmodium protein, unknown function
14	361362.. 361530	3	3′ and 5′ alternative	PF14_0089	Conserved Plasmodium protein, unknown function
14	446307.. 446640	12	Exon skipping	PF14_0108	conserved Plasmodium protein, unknown function
14	448014.. 448158	4	3′ and 5′ alternative	PF14_0778	Conserved Plasmodium membrane protein, unknown function
14	521860.. 522189	5	Exon skipping	PF14_0128	Ubiquitin conjugating enzyme, putative
14	1079304.. 1079501	36	3′ and 5′ alternative	PF14_0253	Conserved Plasmodium membrane protein, unknown function
14	1446211.. 1446527	24	Exon skipping	PF14_0338	Conserved Plasmodium protein, unknown function
14	2016906.. 2017099	36	3′ and 5′ alternative	PF14_0469	Transcription factor IIIb subunit, putative
14	2255846.. 2256076	9	3′ and 5′ alternative	PF14_0526	Conserved Plasmodium protein, unknown function
14	2481102.. 2481202	41	3′ and 5′ alternative	PF14_0581	Apicoplast ribosomal protein S10 precursor, putative
14	2587073.. 2587215	48	3′ and 5′ alternative	PF14_0607	Conserved Plasmodium membrane protein, unknown function
14	2812903.. 2813007	2	3′ and 5′ alternative	PF14_0653	Derlin-2, putative

Overview of alternative splicing events confirmed by RNA-Seq Solexa reads over seven time points used in the study.

### Identification of novel transcripts and expression of pseudogenes

We carefully analysed the expression signals that mapped neither to regions on the genome that correspond to annotated genes nor to their likely UTRs. A region was called positive (i.e. transcriptionally active) if a window of 60 bp was fully covered and had a geometric mean score of 5 or greater. All regions were verified by manual inspection. Using these criteria, we identified 107 novel blood-stage transcripts that were previously unknown (Table S5). These appear to be non-protein coding as no *bona fide* protein-encoding gene prediction is possible for these novel transcripts. With the methods used here we are unable to determine from which strand these transcripts arise. Such non-protein coding transcripts have been described before as mRNA-like non-coding RNAs (mlnc RNA) (Rodriguez *et al.*, 2004; Griffiths-Jones, 2007). There is growing evidence for a large number (> 34 000) of these mlncRNAs in the human genome (Carninci *et al.*, 2005). They are likely to be transcribed by RNA polymerase II but their function in most cases is unknown. One exception is the mammalian Xist mlncRNA transcript that is derived from a pseudogene and plays a vital role in X-dosage compensation (Duret *et al.*, 2006). The length of these transcripts in *P. falciparum* varies from 57 to 8931 bp (median is 918 bp) and the majority appear to be shared with other *Plasmodium* parasites sequenced to date (Table S5). We do not, however, have any experimental evidence to suggest that these orthologous genomic regions are transcribed during the blood stages of infection in other *Plasmodium* parasites. We show the expression profile of one of these novel transcripts in Fig. 3.

There are 81 annotated pseudogenes in the *P. falciparum* 3D7 genome (Tables S4 and S5), of which we detect transcription from 38 in our sequencing data. Of these, 17 were previously found to be expressed in a periodic manner during the IDC (Bozdech *et al.*, 2003) suggesting their recent evolutionary conversion to pseudogenes.

## Discussion

Using high-throughput sequencing (RNA-Seq), this study provides the first in-depth sequencing-based analysis of the *P. falciparum* transcriptome, derived from seven time points from the red blood cell stage of development. Despite the high A+T content of this genome, our data provide near-complete genome coverage of RNA transcripts at single base pair resolution and provide significant improvement to our understanding of the global transcriptome. We identify rare stage-specific alternative splicing events, novel transcripts and low abundance transcripts, and correct existing gene models using the deep read coverage provided by Illumina sequencing. Our high-coverage RNA-Seq data were made possible by the use of a new strategy for depleting high-abundance transcripts (predominantly rRNAs) that would otherwise dominate the sequencing library. This consisted of a combination of an exonuclease digestion, specific for uncapped RNA, followed by oligonucleotide depletion of the most abundant transcripts.

Overall, using the geometric mean coverage for each gene across the seven time points measured, we recapitulate the periodic gene expression patterns previously reported (Bozdech *et al.*, 2003; Le Roch *et al.*, 2003). Using a parallel analysis of the same samples used for deep sequencing by DNA microarrays resulted in a good correlation between these independent methods. The sequencing data provide an expanded IDC transcriptome because we were able to capture the transcript abundance for lower abundance species, resulting in 4871 transcripts detected during the erythrocytic stages of *P. falciparum* life cycle, suggesting that roughly 90% of the genome is transcriptionally active during this stage. Some genes that are known to be expressed in other life cycle stages (such as PFI0185w, expressed in gametocytes, Fig. 3) show no expression during the IDC. In addition to improved sensitivity in detecting transcript expression, these higher resolution RNA-Seq data provide detailed structural data for each of these 4871 transcripts.

We have used the RNA-Seq data for the verification and correction of existing gene models and to create a number of new gene models: 423 existing gene models were corrected and 121 new genes were added to the current annotations. Seventy-five per cent of splice sites were confirmed with at least two read pairs. For all novel transcripts detected here, we also report the gene expression patterns during parasite development in red blood cells. Future improvement in technology such as increased read length, larger insert sizes and strand-specific reads will dramatically improve overall mapability, our ability to analyse splice sites and UTRs, and possibly to identify anti-sense transcripts. RNA-Seq of additional life cycle stages such as gametocytes, oocytes, sporozoites and liver stages is also expected to unravel stage-specific alternative splicing events and add more new transcripts.

Our results identified only 84 alternative splice sites, which is perhaps unexpected, because in many higher eukaryotes over half of transcripts are alternatively spliced (Zavolan and van Nimwegen, 2006). A recent study seeking to identify alternative splicing in late-stage schizont genes and in gametocytes found alternative splicing for 16% of the 88 open reading frames characterized (Iriko *et al.*, 2009), but found only two events of alternative splicing in the blood stages. While we detected 84 alternative splicing events, we anticipate that with increased sequencing read length, it will become easier to identify bridging reads and thus enhance our current understanding of the role of alternative splicing. A major question that remains unanswered is what mechanisms are being used to regulate the production of alternative splice forms and are they functional?

The true power of RNA-Seq is that from one experiment, the full transcript, from transcription start site to polyadenylation signal, will be captured. Unfortunately, due to the high A+T content of the *P. falciparum* genome as well as the prevalence of low complexity regions, this study falls short of providing significant information about non-coding regions. Undoubtedly, on a per-gene basis, there is a lot of information to be gained, but this is difficult to generalize computationally across the genome.

The results of this work demonstrate how RNA-Seq can further our understanding of blood-stage transcription, including insights into post-transcriptional events. Given that our understanding of transcriptional regulation remains poor for *P. falciparum*, accurate gene models of RNA transcripts are essential. Ongoing efforts are underway to extend sequencing-based transcriptome analyses to other *Plasmodium* life cycle stages including gametocytogenesis, mosquito development and liver-stage development. Of great interest is the sequencing of transcripts from individual patient isolates to continue to address the degree to which there are differences between the *in vivo* and *in vitro* transcriptome of this parasite as has been previously suggested (Daily *et al.*, 2007; Lemieux *et al.*, 2009).

## Experimental procedures

### RNA preparation

Highly synchronous *P. falciparum* cultures were attained by growing 50 ml cultures of parasites in RPMI 1640 culture medium (with standard supplements) and using 5% sorbitol to select for ring-stage parasites by standard methods (Trager and Jensen, 1976; Lambros and Vanderberg, 1979). Initially, one 50 ml culture (5% parasitaemia) was synchronized 2 h post invasion and subsequently at 10 h, following re-invasion, newly formed rings were again selected for with two sorbitol treatments. The cultures were then expanded to sufficient culture flasks at a final parasitaemia of 10%. Total RNA from *P. falciparum* was isolated as described previously (Bozdech *et al.*, 2003). Briefly, a TRIZOL extraction was performed followed by an overnight sodium acetate in isopropanol precipitation, with a final 70% ethanol wash.

### Depletion protocol

For custom depletion of *P. falciparum* rRNAs and high-abundance transcripts, 500 µl of streptavidin beads (Dynabeads, Invitrogen) was washed (10 mM Tris-HCl, pH 7.5, 1.0 mM EDTA, 2.0 M NaCl) three times and resuspended in 200 µl of the same buffer plus 200 µl of a mix of biotin-labelled oligonucleotides (100 pmol of each) (Table S1) and incubated at 37°C for 10 min. The beads were then washed three times with 1 mM sodium citrate, pH 6.4. 25 µg of total RNA was then incubated for 10 min at 65°C in a final volume of 200 µl 1 mM sodium citrate, 0.4 M guanidine, 1 µl RNAseAway. After cooling to room temperature for 10 min, the bound RNA was removed by magnetic purification of the beads. The free RNA was cleaned using a Zymo RNA column. For Terminator™ 5′-Phosphate-Dependent Exonuclease (Epicentre) reactions, the manufacturer's recommendations were followed. Briefly, 25 µg of *P. falciparum* total RNA was incubated with 2 µl of exonuclease in a final volume of 40 µl for 1 h. After 1 h the reaction was stopped with 2 µl 0.1 mM EDTA, pH 8.0 and immediately added to the biotin-labelled immobilized beads (see above). For *P. falciparum*, cDNA synthesis and purification, we used a combination of random oligonucleotide and oligo(dT) primers as previously published (Bozdech *et al.*, 2003).

### Sample preparation Solexa

Sequencing libraries for the Illumina GA II platform were constructed by shearing the enriched cDNA by nebulisation (35 psi, 6 min) followed by end-repair with Klenow polymerase, T4 DNA polymerase and T4 polynucleotide kinase (to blunt-end the DNA fragments). A single 3′ adenosine moiety was added to the cDNA using Klenow exo- and dATP. The Illumina adapters (containing primer sites for sequencing and flowcell surface annealing) were ligated onto the repaired ends on the cDNA and gel-electrophoresis was used to separate library DNA fragments from unligated adapters by selecting cDNA fragments between 200 and 250 bp in size. Ligated cDNA fragments were recovered following gel extraction at room temperature to ensure representation of AT-rich sequences. Libraries were amplified by 18 cycles of PCR with Phusion DNA polymerase (Finnzymes Reagents).

The efficacy of each stage of library construction was ascertained in a quality control step that involved measuring the adapter-cDNA on an Agilent DNA 1000 chip. Sequencing libraries diluted to 2 nM were denatured with sodium hydroxide and diluted to 3.5 pM in hybridisation buffer for loading onto a single lane of an Illumina GA flowcell. Cluster formation, primer hybridisation and single-end (or read pairs) of either 37 or 54 sequencing cycles were performed using proprietary reagents according to manufacturer's recommended protocol (https://icom.illumina.com/).

As we used both 37 and 54 bp read pairs in this experiment, we compared the RNA-Seq results from the sample sequenced both ways. The correlation between the geometric mean (defined as the exponential of the product of the natural logarithm of the coverage of *n* unique bases of a given CDS) was between 0.98 and 1.0. In the calculation of this mean, non-unique regions of the gene were ignored. If one read of a mate pair can be placed in a non-unique region, this coverage is not counted.

### Processing of Illumina data

Figure 1 describes the read-processing pipeline used in this study. We used SSAHA2 (Ning *et al.*, 2001) and SSAHA_pileup to align the Illumina reads against the *P. falciparum* 3D7 (version 2.1.4) reference genome. We also used MAQ (Li *et al.*, 2008) to investigate the success of the different depletion strategies because MAQ randomly places repetitive reads such as those aligning to rRNA sequences. In the SSAHA2 mapping, we only included reads where one end of the pair aligned uniquely to the genome and the distance between the pairs was within the expected insert size. The output of SSAHA_pileup was used to create the coverage plots over the genome. We generated uniqueness plots for all possible windows of 30 or 50 bp over the genome. All repeat regions longer than the read length in a gene are ignored for the expression calculation. To measure the expression levels of each gene, we used the geometric mean.

### DNA microarray analysis

For each time point, 12 µg of total RNA was prepared for hybridization by indirect amino-allyl cDNA labelling as previously described (Bozdech *et al.*, 2003). A pool of 3D7 total RNA from all IDC stages was utilized as the reference sample. Array hybridizations were performed as described using a recently designed new-generation *P. falciparum*-specific long oligonucleotide DNA microarray (Hu *et al.*, 2007). The arrays were scanned using an Axon 4200A scanner and images analysed using Axon GenePix software (Axon Instruments, Union City, CA, USA). Due to technical reasons, we were not able to successfully attain DNA microarray hybridization data for the 40 h time point.

### Correlation between RNA-Seq and DNA microarray results

The IDC maps (Fig. 2) were created in R with the heatmap command. The order for the genes was taken from Llinas *et al.* (2006). The correlation with the microarray was also calculated in R using the Pearson correlation. To compare the correlation between the different read lengths, we used the logarithm of the geometric mean of each CDS. For the comparison with the array data, we used the expression of CDS of the 54 bp reads and compared it with the Cy5 values of the array data. If for a given gene all time points of the array were not available, the gene was ignored for the analysis.

### Splice site determination

To find new splice sites or to confirm existing splice sites, we used bridging reads. These are reads that would map over a splice site in the nucleotide sequence of the gene, but in the alignment output will split align to two different regions of a chromosome, as they are mapping to two different exons. To find new splice sites, we look for reads that do not map entirely on the CDS. We then required that these possible splice site confirming reads map with no more than one mismatch to an existing CDS or a new exon and each part of the read must have a match of at least 13 bp.

The non-mapping regions of the read provide information about where another exon is beginning. Therefore, those reads are remapped with SSAHA2 using very sensitive parameters (-seeds 1 -score 10 -cmatch 9 -ckmer 6 -kmer 9 -cut 2000000 -skip 1), generating multiple partial hits. A read identifies a putative new splice site if two partial hits map to the same chromosome and strand, their distance is less than 10 000 bp, and the genomic sequence contains a splice donor (GT) and acceptor (AG).

To define alternative splicing (Table 3) we mapped all the reads against gene models containing the new splice site and counted how many reads confirmed the new splice site. We required at least two confirming reads to call the potential new splice site.

### UTR coverage

To estimate the coverage of UTR regions, we analysed the 1 kb upstream and downstream regions of each CDS (providing this region did not overlap with another gene). We counted the number of mapping reads and the amount of covered bases for each 5′ and 3′ region.

### RT-PCR verification of RNA-Seq results

Twenty micrograms of mixed asexual stage total RNA prepared according to the Trizol method was DNase treated and cleaned up using a QIAgen RNeasy column before reverse transcription using Superscript III (Invitrogen) with a 1:1 mixture of poly-dT : poly-dN according to manufacturer's protocol. One hundred nanograms of cDNA was used in each PCR reaction and run for 35 cycles. Primers used are listed in Table S6.
