# Supplementary material for: New insights into the blood-stage transcriptome of Plasmodium falciparum using RNA-Seq
Source: Mol Microbiol. 2010 Feb 5;76(1):12–24. doi: 10.1111/j.1365-2958.2009.07026.x (PMC2859250; doi:10.1111/j.1365-2958.2009.07026.x)
Supplement: Supplementary file 1 [file mmi0076-0012-SD1.pdf]

Figure S1:

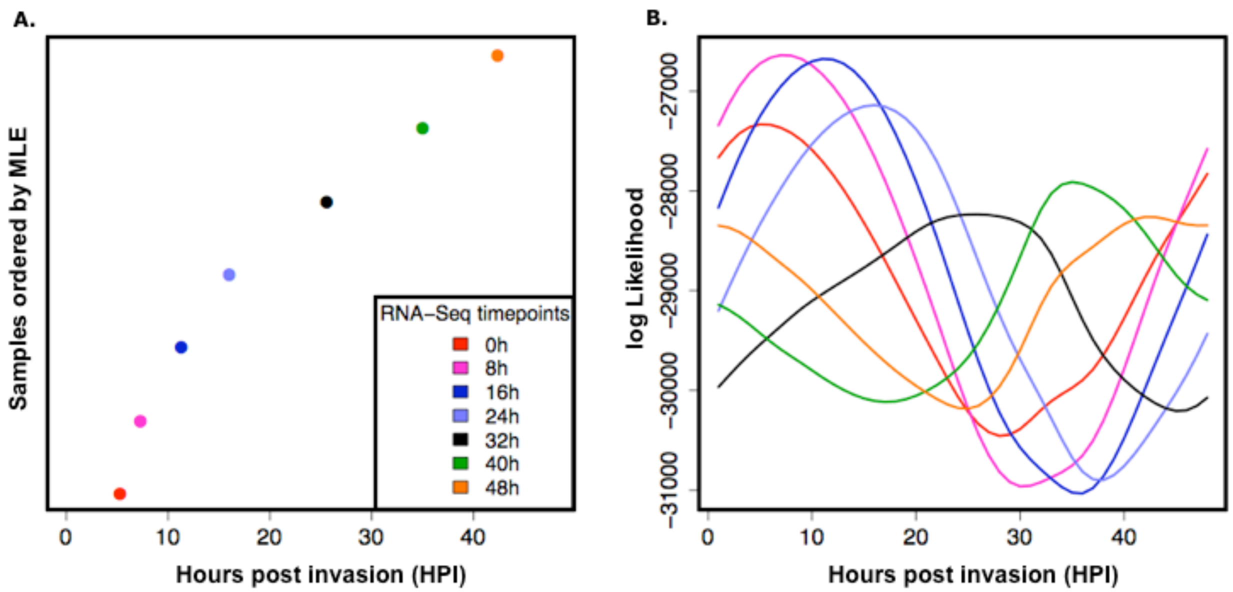

Figure S1: Maximum likelihood estimates (MLE) (A) and log-likelihood curves of temporal progression (B) as computed by the method in Lemieux et al. 2009 (Lemieux *et al.*, 2009). To produce these estimates, a likelihood function is obtained for each gene by evaluating the probability of the observed expression value at each time value of a given reference set (in this case, the hourly expression profiles of (Bozdech *et al.*, 2003)). The log-likelihood for the entire sample is then computed by summing the log-likelihood curves of individual genes. The maximum likelihood estimate for hours post invasion is the maximum of sample log-likelihood curve.

Lemieux, J. E., N. Gomez-Escobar, A. Feller, C. Carret, A. Amambua-Ngwa, R. Pinches, F. Day, S. A. Kyes, D. J. Conway, C. C. Holmes & C. I. Newbold, (2009) Statistical estimation of cell-cycle progression and lineage commitment in Plasmodium falciparum reveals a homogeneous pattern of transcription in ex vivo culture. *Proc Natl Acad Sci U S A* **106**: 7559-7564.

Bozdech, Z., M. Llinas, B. L. Pulliam, E. D. Wong, J. Zhu & J. L. DeRisi, (2003) The Transcriptome of the Intraerythrocytic Developmental Cycle of Plasmodium falciparum. *PLoS Biol* **1**: E5.

Figure S2:

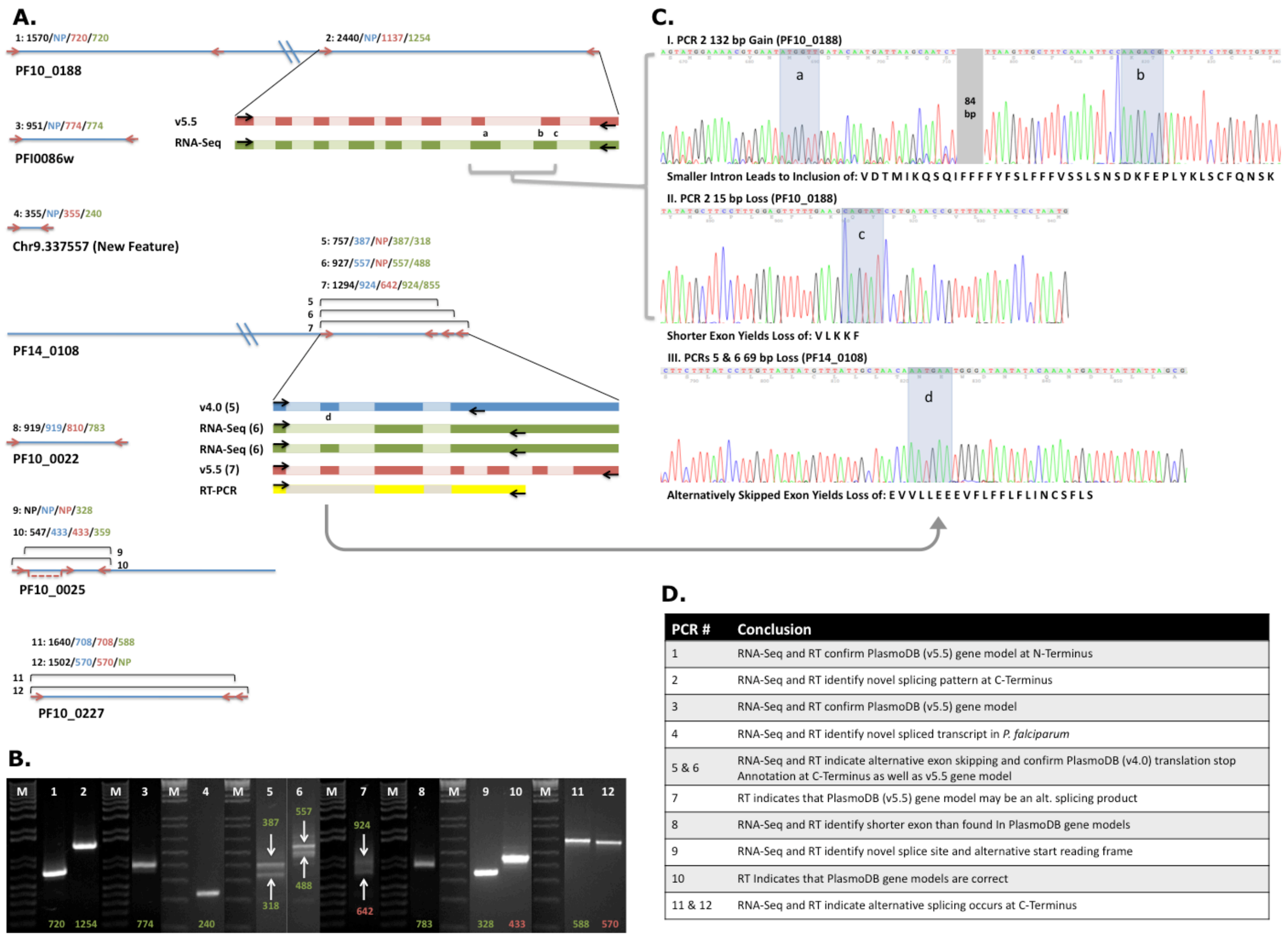

PCR #	Conclusion
1	RNA-Seq and RT confirm PlasmoDB (v5.5) gene model at N-Terminus
2	RNA-Seq and RT identify novel splicing pattern at C-Terminus
3	RNA-Seq and RT confirm PlasmoDB (v5.5) gene model
4	RNA-Seq and RT identify novel spliced transcript in *P. falciparum*
5 & 6	RNA-Seq and RT indicate alternative exon skipping and confirm PlasmoDB (v4.0) translation stop Annotation at C-Terminus as well as v5.5 gene model
7	RT indicates that PlasmoDB (v5.5) gene model may be an alt. splicing product
8	RNA-Seq and RT identify shorter exon than found In PlasmoDB gene models
9	RNA-Seq and RT identify novel splice site and alternative start reading frame
10	RT Indicates that PlasmoDB gene models are correct
11 & 12	RNA-Seq and RT indicate alternative splicing occurs at C-Terminus

Figure S2. RT-PCR verification of RNA-Seq predictions. A. Chromosomal model of seven genes used to verify RNA-Seq predictions. In each case, a blue horizontal line indicates the predicted chromosomal size for each gene as derived from the PlasmoDB v5.5 translation start to translation stop. In the case of the newly found feature Chr9.337557, the length of the chromosomal segment corresponds to gene boundaries predicted by RNA-Seq. Hatch marks indicate a break in the gene model for long genes. Red arrows indicate the positions of primers used to verify new gene models. Above the gene, the expected DNA sizes for RT-PCR reactions are listed for reference. The four sizes indicate the expected PCR product size for genomic DNA amplification (black), and complementary DNA (cDNA) amplification based on PlasmoDB v4.0 (Blue), PlasmoDB v5.5 (Red) or RNA-Seq (Green) gene models. In cases where primers are outside of the predicted open reading frame or bridge putative introns, no product (NP) is predicted. In cases where primers fall in putative 3’ UTRs we assumed that the UTR will extend to the position of the primer. The insets below PF10_0188 and PF14_0108 show the various predicted gene models with exons in bold color and introns in shaded

color. Distances for each gene are approximate. **B.** RT-PCR results. In each case, the size of the PCR product is indicated at the bottom of the lane (marked with the number of the PCR reaction) in a color corresponding to the three gene model predictions. RT-PCR was carried out according to standard procedures. Briefly, 20 μg of mixed asexual stage total RNA prepared according to the Trizol method was DNase treated and cleaned on a QIAgen RNeasy column before reverse transcription using Superscript III reverse transcriptase (Invitrogen) and a 1:1 mixture of poly-dT:poly-dN according to manufacturer's protocol. 100 ng of cDNA were used in each PCR reaction. PCR products expected to be less than 600 bp were run on a 2% agarose gel while PCR products expected to be greater than 600 bp were run on a 1% agarose gel. M: 1kb DNA ladder. For PF10_0188, PF10_0022, and PF14_0108, the RT-PCR products were confirmed by dideoxy sequencing. PF10_0188 was indeed correct as predicted by RNA-Seq at 783 bp (data not shown). PF10_0188 and PF14_0108, see in C. **C.** Dideoxy sequencing of gel-purified fragments verifies novel spliced transcripts. PCR 2,5, and 6 products were gel purified and sequenced. In I, new 5' and 3' splice sites lead to a smaller C-terminal intron. Highlighted regions (a and b) denote previous splice sites. The in-frame translated sequence of the additional 132 bp are shown below and are predicted to form an alpha-helix according to JPred3 (Cole *et al.*, 2008). In II, a shortened downstream exon leads to the loss of five amino acids (splice junction, c highlighted). In III, an alternatively skipped exon leads to the loss of a 23 amino acid sequence predicted to form an alpha-helix according to Jpred3 (splice junction, d highlighted). **D.** Table summarizing the conclusions to be drawn from RT-PCR verifications. MP: massively parallel, DD: dideoxy.

Cole, C., J. D. Barber & G. J. Barton, (2008) The Jpred 3 secondary structure prediction server. *Nucleic Acids Res* **36**: W197-201.
