# Supplementary material for: New insights into the blood-stage transcriptome of Plasmodium falciparum using RNA-Seq
Source: Mol Microbiol. 2010 Feb 5;76(1):12–24. doi: 10.1111/j.1365-2958.2009.07026.x (PMC2859250; doi:10.1111/j.1365-2958.2009.07026.x)
Supplement: Supplementary file 7 [file mmi0076-0012-SD7.doc]

**Table S6:** Primers for RT-PCR Verification of RNA-Seq Predictions

| **Name** | **Sequence (5’ – 3’)** | **PCR #s** |
| --- | --- | --- |
| PF10_0188_For_N | atgaataaagctatggatttaatagac | 1 |
| PF10_0188_Rev_N | GGACACATTGTCAAAAGACACAAAGG | 1 |
| PF10_0188_For_C | GGATGCAATCAGTTTAACACAGCTG | 2 |
| PF10_0188_Rev_C | TCATTTTGCACTCCACATTCCCAAAC | 2 |
| PFI0086w_For | atgtttatgaaaaaatttatgaaggcaaac | 3 |
| PFI0086w_Rev | ttatgtaaaatatatcttttttttttttccacaatgacg | 3 |
| Chr9.337557_For | ATGAGTAATGATCAGGATTTAAAAAGTTC | 4 |
| Chr9.337557_Rev | TTAATTTGCTGATAAGTTTTTTTTTTTAAGTATC | 4 |
| PF14_0108_For | atggaaatcagactttcttctttatcc | 5-7 |
| PF14_0108_Rev_Old | ttatgaaccttgaattaccttctcac | 5 |
| PF14_0108_Rev_Seq | ttaatagactacctcatgagcaacag | 6 |
| PF14_0108_Rev_New | ttatttttttgtacatttaaacttattaaaac | 7 |
| PF10_0022_For | ATGGATAAGACAAATTATAGTATAAAGAA | 8 |
| PF10_0022_Rev | TTATAATCTCTGATTTAATGCCTTTTTTA | 8 |
| PF10_0025_For_Old | ATGGAAAACATAATAAACAAGAAGAATAC | 9 |
| PF10_0025_For_Seq | ATGGGGGAAATATATACTTATAATGG | 10 |
| PF10_0025_Rev | CCGCACTAGTTCCTTGCTCATTTG | 9,10 |
| PF10_0227_For | atggaaacttatttagtagatttattgag | 11,12 |
| PF10_0227_Rev_Old | TTATTTATTTATTACTTGACATATATAATTTGGC | 11 |
| PF10_0027_Rev_Seq | TTACGTGACATTTTTTGGACTTTCTAC | 12 |
